# Supplementary material for: Calcium Binds to Transthyretin with Low Affinity
Source: Biomolecules. 2022 Aug 2;12(8):1066. doi: 10.3390/biom12081066 (PMC9406000; doi:10.3390/biom12081066)
Supplement: Supplementary file 1 [file biomolecules-12-01066-s001.zip › biomolecules-1846284-supplementary.pdf]

Supplementary material for

Calcium Binds to Transthyretin with Low Affinity

Cristina Cantarutti [1,2], Maria Chiara Mimmi [3], Guglielmo Verona [4], Walter Mandaliti [1], Graham W. Taylor [4], P. Patrizia Mangione [3,4], Sofia Giorgetti [2,3], Vittorio Bellotti [2,4,5] and Alessandra Corazza [1,2,*]

1 Department of Medicine, University of Udine, 33100 Udine, Italy; cristina.cantarutti@uniud.it (C.C.); walter.mandaliti@uniud.it (W.M.)
2 Istituto Nazionale Biostrutture e Biosistemi, 0013 Rome, Italy; sofia.giorgetti@unipv.it (S.G.); v.bellotti@ucl.ac.uk (V.B.)
3 Department of Molecular Medicine, Institute of Biochemistry, University of Pavia, 27100 Pavia, Italy; chiara.mimmi@unipv.it (M.C.M.), palma.mangione@unipv.it (P.P.M.)
4 Wolfson Drug Discovery Unit, Centre for Amyloidosis and Acute Phase Proteins, Division of Medicine, University College London, NW3 2PF London, UK; g.verona@ucl.ac.uk (G.V.); graham.taylor@ucl.ac.uk (G.W.T.)
5 Scientific Direction, Fondazione IRCCS Policlinico San Matteo, 27100 Pavia, Italy
* Correspondence: alessandra.corazza@uniud.it

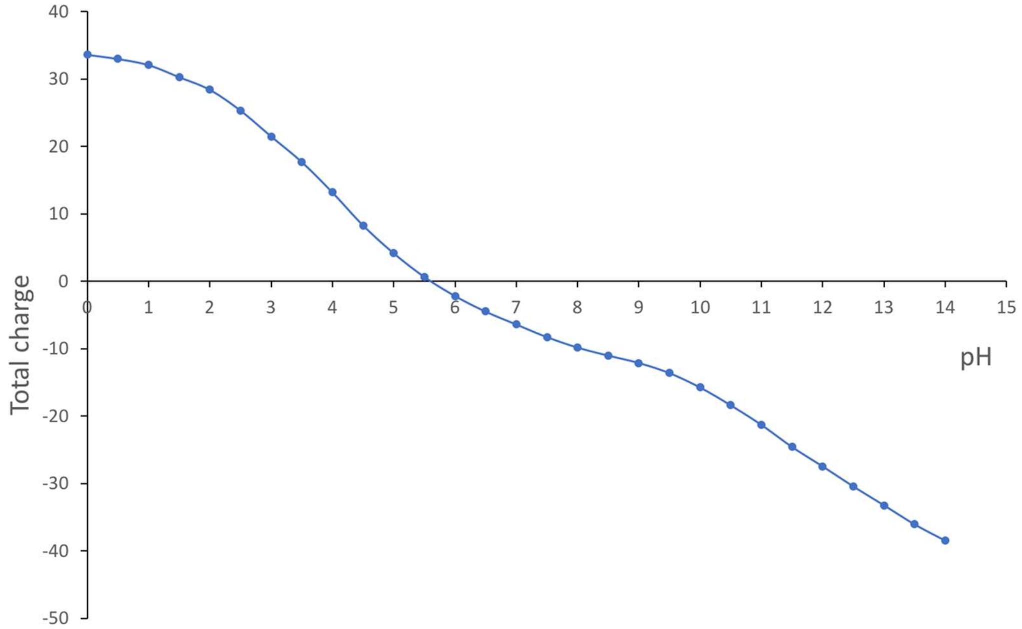

Figure S1. TTR charge as a function of pH.

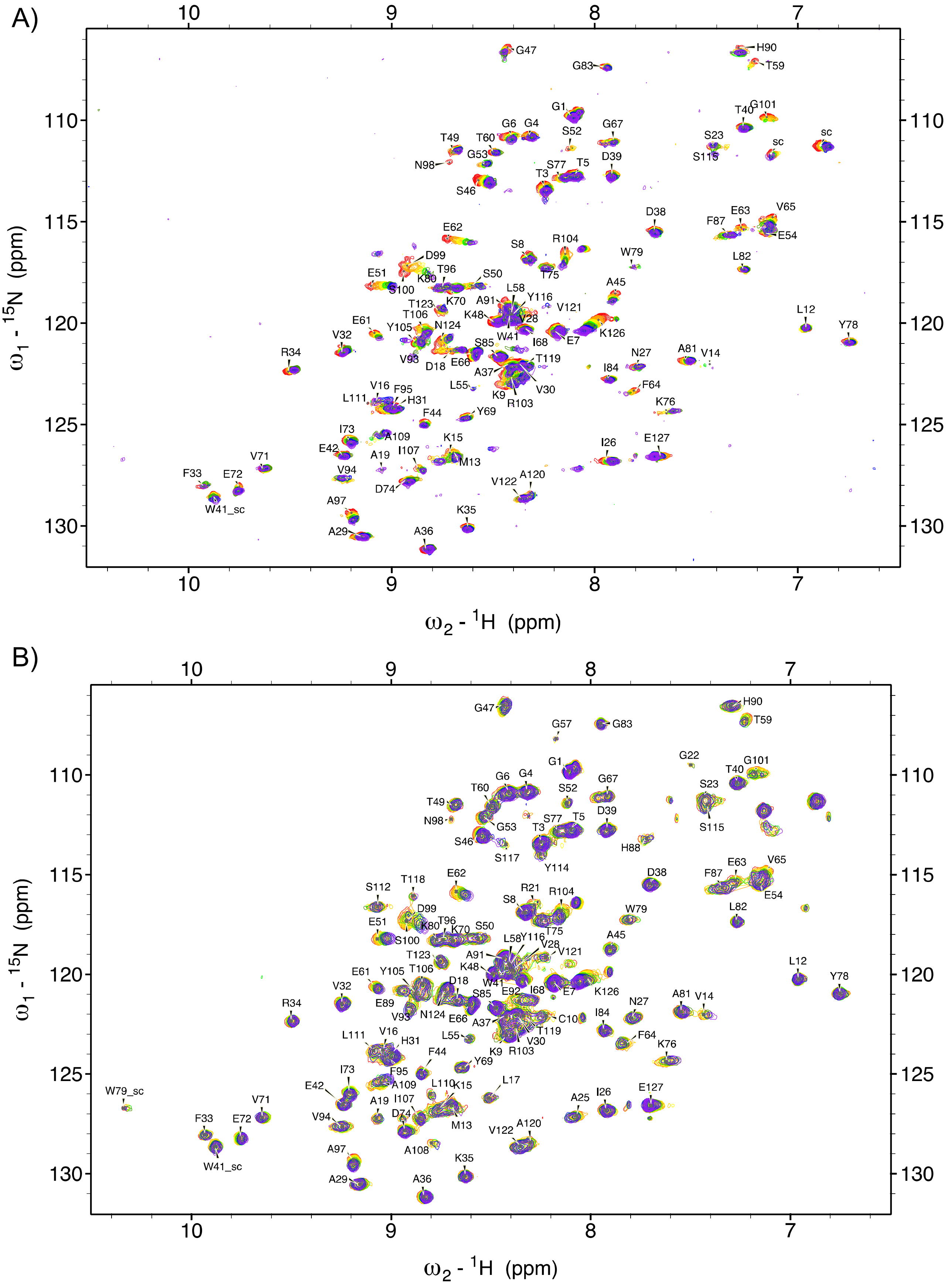

Figure S2. Overlay of 2D [^{1}H, ^{15}N] TROSY spectra of TTR acquired at 700 MHz recorded at increasing calcium concentration at pH 7.4 without (**A**) and with 154 mM NaCl (**B**). The color code for panel A is:0mM red, 5 mM orange, 10mM yellow, 20mM green, 40mM blue, 60mM purple. The color code for panel B is: 0 mM red, 1.3 mM orange, 4.6 mM gold, 7.9 mM yellow, 14.5 mM green, 24.3 mM chartreuse green, 37.3 mM blue and 62.7 mM purple.

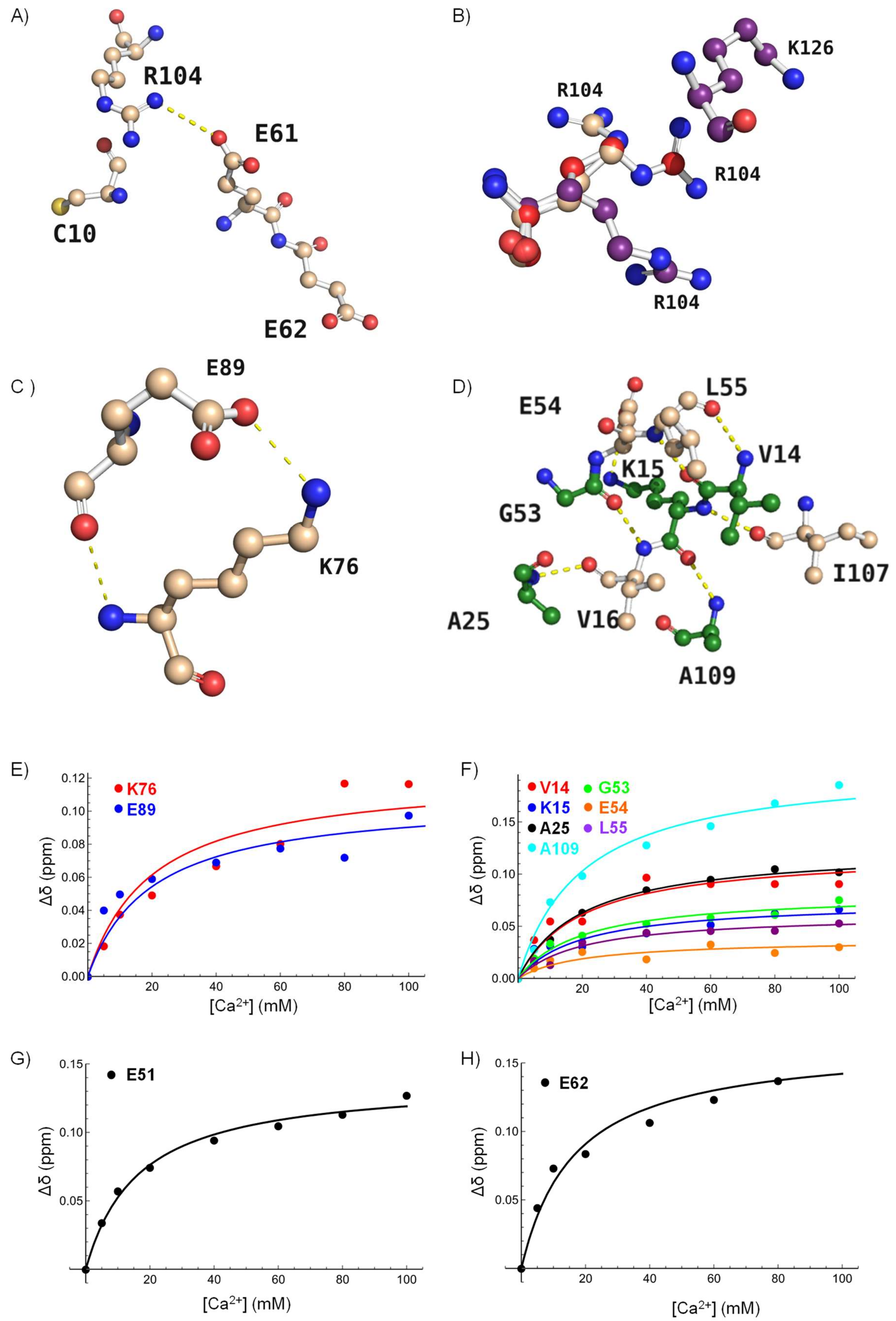

Figure S3. (**A**) C10, R104 and E61 residues taken from 5CN3 TTR structure. (**B**) Position of R104 side chain with respect to K126 in 5CN3 (beige), 1TTA (purple) and 4N85 (red) structures. (**C**) H bonds between E89 and K76 in 5CN3 structure. (**D**) H bond network in 5CN3 structure connecting significantly shifting residues in presence of calcium (coloured in green). (**E-H**) Chemical shift perturbation as a function of Ca^{2+} concentration recorded at pH 6.5 and fitted with equation [1] (see main text) for residues with $\Delta\delta \geq$ average $+2\sigma$ belonging to putative sites 3-6.

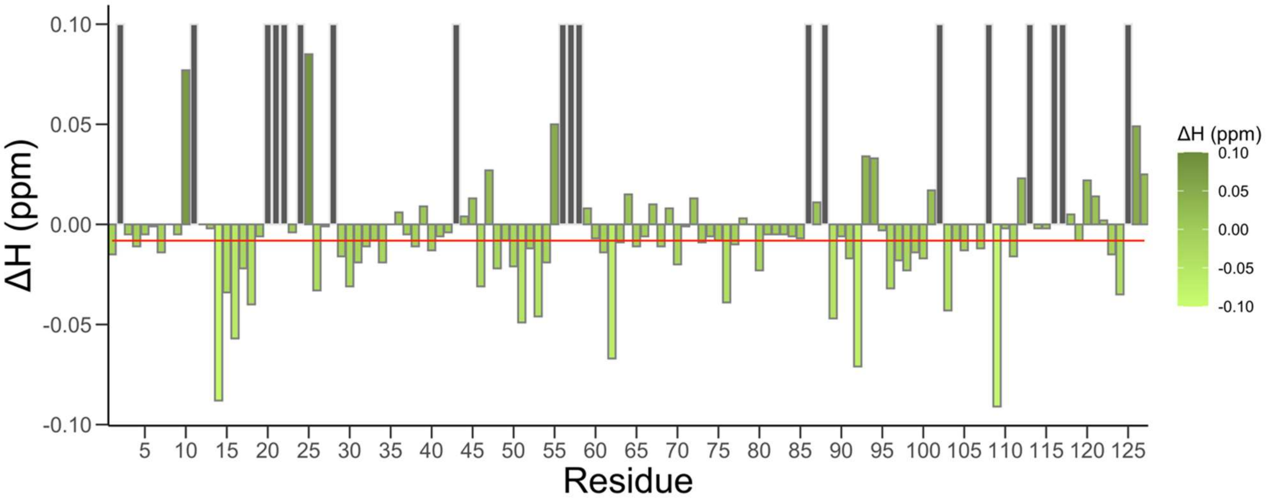

Figure S4. Bar plot of TTR H^N chemical shift variation with 150 mM NaCl. Gray bars indicate prolines and residues that could not be followed during titration. The H^N chemical shift variation observed in the presence of NaCl is depicted in a light-dark green gradient. The red line corresponds to the average value.

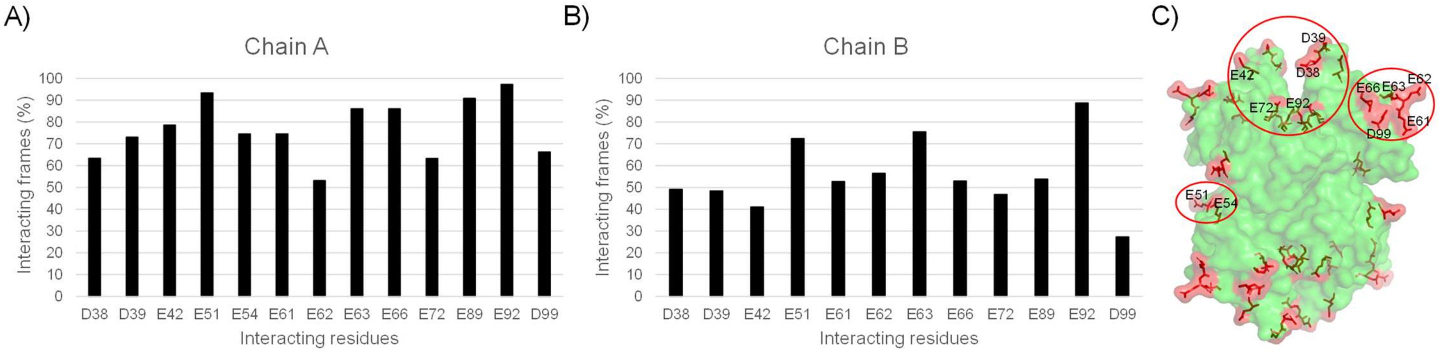

Figure S5. Persistency of electrostatic interactions between Ca^{2+} and TTR residues obtained from 15 ns MD simulations for protein chain A (**A**) and chain B (**B**). (**C**) The interacting residues are coloured in red on the protein structure (pdb: 5cn3) and the three interacting patches are highlighted by red circles.
